# Postal survey of physicians and laboratories: Practices and perceptions of molecular oncology testing

**DOI:** 10.1186/1472-6963-9-131

**Published:** 2009-07-30

**Authors:** Fiona A Miller, Paul Krueger, Robert J Christensen, Catherine Ahern, Ronald F Carter, Suzanne Kamel-Reid

**Affiliations:** 1 Department of Health Policy, Management and Evaluation, University of Toronto, Toronto, Canada; 2 Department of Family and Community Medicine, University of Toronto, Toronto, Canada; 3 Department of Pathology and Molecular Medicine, McMaster University, Hamilton, Canada; 4 Department of Lab Medicine and Pathobiology, University of Toronto; Department of Pathology, University Health Network, Toronto, Canada

## Abstract

**Background:**

Molecular oncology testing (MOT) to detect genomic alterations underlying cancer holds promise for improved cancer care. Yet knowledge limitations regarding the delivery of testing services may constrain the translation of scientific advancements into effective health care.

**Methods:**

We conducted a cross-sectional, self-administered, postal survey of active cancer physicians in Ontario, Canada (N = 611) likely to order MOT, and cancer laboratories (N = 99) likely to refer (i.e., referring laboratories) or conduct (i.e., testing laboratories) MOT in 2006, to assess respondents' perceptions of the importance and accessibility of MOT and their preparedness to provide it.

**Results:**

54% of physicians, 63% of testing laboratories and 60% of referring laboratories responded. Most perceived MOT to be important for treatment, diagnosis or prognosis now, and in 5 years (61% – 100%). Yet only 45% of physicians, 59% of testing labs and 53% of referring labs agreed that patients in their region were receiving MOT that is indicated as a standard of care. Physicians and laboratories perceived various barriers to providing MOT, including, among 70% of physicians, a lack of clear guidelines regarding clinical indications, and among laboratories, a lack of funding (73% – 100%). Testing laboratories were confident of their ability to determine whether and which MOT was indicated (77% and 82% respectively), and perceived that key elements of formal and continuing education were helpful (75% – 100%). By contrast, minorities of physicians were confident of their ability to assess whether and which MOT was indicated (46% and 34% respectively), and while majorities considered various continuing educational resources helpful (68% – 75%), only minorities considered key elements of formal education helpful in preparing for MOT (17% – 43%).

**Conclusion:**

Physicians and laboratory professionals were enthusiastic about the value of MOT for cancer care but most did not believe patients were gaining adequate access to clinically necessary testing. Further, our results suggest that many were ill equipped as individual stakeholders, or as a coordinated system of referral and interpretation, to provide MOT. These challenges should inspire educational, training and other interventions to ensure that developments in molecular oncology can result in optimal cancer care.

## Background

Personalized or 'targeted' oncology is made possible by scientific developments identifying the genetic and genomic alterations underlying cancer. Some of these alterations are hereditary in nature and have particular relevance for individuals with high-risk family histories, but most are *acquired *genomic alterations with relevance to all forms of malignancy. Increased understanding of the significance of acquired genomic alterations in malignancy holds considerable promise for improved cancer care but knowledge about the delivery of associated laboratory services is limited, constraining the ability to translate scientific advancements into improvements in cancer care [1-6].

Molecular oncology testing for acquired genomic alterations in cancer (MOT) can provide more refined diagnoses and more accurate prognoses, contributing to the molecular classification of malignancies. Indeed, some sporadic cancers are already defined and named according to their genetic characteristics (e.g., for acute myeloid leukemia, (AML) M3 with t(15;17), AML with t(8;21), or AML with inv16); a trend that is expected to increase[7,8]. However, molecular oncology testing is important for more than disease classification. Decisions about therapy are also informed by such testing, with sub-types and prognostic markers sometimes used to guide more aggressive approaches to treatment[9,10]. Further, molecular oncology testing has special relevance where specific biomarkers can guide targeted therapy. For example, clinical guidelines recommend treatment with Trastuzumab in patients with early stage breast cancer in which the human epidermal growth factor-like receptor No 2 (HER-2) is overexpressed[11]. Thus, laboratory testing, including MOT, is necessary to assess the status of the HER2 oncogene in most breast cancer patients, to guide the appropriate use of this expensive therapeutic [12-15]. More recently, the use of biological therapeutics such as Panitumumab for metastatic colorectal carcinoma require MOT for detection of mutations in the KRAS gene before patients are eligible for treatment with this antibody[16].

To date, research on the delivery of genetic testing services has focused on testing for germline changes associated with hereditary disease [17-19], or molecular genetic testing more generally [20-22]. Much research has been motivated by policy interest in genetic testing, where such testing is defined broadly to include heritable or acquired genetic changes[23]. For example, a recent "horizon scan" review by the US Agency for Healthcare Research and Quality (AHRQ) adopts such a definition in identifying the genetic tests relevant to cancer care that are both clinically available and under development . Yet while attention to the effective provision of health services for hereditary disease is needed, and will increase as germline genetic testing expands with the development of high throughput technologies, the scope and significance of molecular oncology testing for *sporadic *disease still warrants focused attention. Indeed, unlike the relatively small proportion of cancers that are hereditary and for which germline genetic testing is relevant, molecular oncology testing for the acquired genomic changes underlying sporadic cancer is potentially relevant to *all *forms of malignancy. Further, the delivery of these services is unlikely to require a genetic modality of care, where testing is offered alongside genetic counselling and within specialized genetic clinics[24].

The effective and appropriate delivery of molecular oncology testing for sporadic cancer relies on a coordinated system of referral and interpretation involving both physicians and laboratory professionals. In Ontario, Canada, appropriate referral is a responsibility of physicians who request laboratory analysis, and those cancer laboratories that receive patient samples for testing but lack the capacity to conduct MOT. Such "referral laboratories" must submit relevant samples to the smaller number of "testing laboratories" in the province with the professional and technological credentials to conduct MOT. These testing laboratories must then conduct and report on relevant molecular oncology analyses, providing a report to the referring physician that can inform clinical decision-making.

Access to most laboratory cancer services is free to residents of Ontario at the point of care, as part of the publicly-funded Medicare system. While this minimizes the significance of financial constraints for the patient, financial constraints are not irrelevant to involved health professionals. Little of the provincial funding for complex test technologies is allotted per test, or for a volume of testing. Instead, hospital-based laboratories incorporate the costs of much of the relevant testing within capped global budgets. Decisions about which tests to provide or adopt are therefore constrained, potentially leading to delays in the uptake of relevant tests. Further, physicians may be aware of tests that they deem clinically useful that are *not *provided through provincially funded labs. While the province has a pre-approval system for the public coverage of testing conducted out-of-province, it can be cumbersome and time consuming; further, physicians seeking more rapid access to relevant tests would pass on any associated costs to patients. Meanwhile, some physicians might be uncertain about the extent to which relevant tests would be made available to patients free at the point of care.

Discovery research on genomic technologies with application to cancer care is developing at a rapid pace. Yet translational research to move validated discoveries into health care practice, through research on the delivery, dissemination and diffusion of innovations – dubbed "T3" by Khoury and colleagues – has fallen behind[25]. A recent publication by Wideroff and colleagues reports the deliberations of a US National Cancer Institute workshop that examined the state of health services research on these and related technologies, and called for a comprehensive health services research agenda to address existing deficiencies, including research on utilization, access and provider preferences[1]. In the absence of a body of health services research on MOT, we conducted a postal survey examining the attitudes and practices of laboratories that perform MOT (i.e., testing laboratories), laboratories that refer samples for MOT (i.e., referring laboratories), and physicians who order or utilize MOT results in Ontario. The study was designed to be exploratory and descriptive – to identify the attitudes and reported practices of key stakeholder groups, provide a baseline data set, and suggest areas of concern for future hypothesis-driven research. Because of the importance of coordinated approaches to referral and testing for the appropriate and effective delivery of these laboratory services, we were especially interested to identify differences in attitudes between physicians, referring laboratories and testing laboratories. In addition, because we expected that physicians might vary considerably in their ability to make effective use of MOT services, we were interested to explore whether certain physician characteristics (e.g., being a specialist provider, or provider of care for the hematological malignancies, etc.) were consistently associated with reported attitudes, and with the anticipated valence.

## Methods

With ethics approval from McMaster University's Research Ethics Board, we conducted a cross-sectional, self-administered postal survey of physicians and laboratories in Ontario, Canada in 2006. Questionnaires were sent to 611 cancer physicians in Ontario who were likely to order or use MOT. This included physicians identified as oncologists, and other physicians with a medical specialty or interest in oncology as defined by MDSelect, the Canadian Medical Directory, made available for purchase by the Canadian Medical Association (e.g., hematologists or gynecologists with a sub-specialty in oncology, family physicians with a medical interest in oncology, etc.).

In addition, we sent questionnaires to 99 hospital-based, public laboratories in Ontario that were likely to (1) conduct MOT, hereinafter testing laboratories; or (2) receive cancer samples where MOT testing might be indicated and, in the absence of on-site capacity, refer these samples to testing laboratories for relevant analyses, hereinafter referring laboratories. Relevant sites included genetics, hematology and pathology laboratories involved in assessing cancer samples. We identified potential laboratories through publicly available lists of Ontario hospitals, supplemented by telephone contact to clarify whether MOT was referred out or conducted on-site, and to identify one person (laboratory director, medical director, technical director, site supervisor or senior pathologist/hematologist) to whom a questionnaire regarding the laboratory should be directed.

Up to five contacts were made with potential physician or laboratory professional respondents over a 7 week period, following a modified version of the Dillman Tailored Design method[26]. The first mailing was a notice letter to apprise potential respondents of the study. The second mailing included a study package (cover letter, numbered questionnaire, postage paid return envelope) and financial incentive ($5 gift certificate at a popular coffee shop). The third mailing to everyone was a thank you/reminder letter. The fourth mailing of a study package was sent to non-respondents. For remaining non-respondents, a fifth and final study package was sent by courier.

The questionnaire asked respondents about their involvement with and attitudes toward molecular oncology testing, which we explicitly and repeatedly defined as testing for *acquired *genetic changes in solid tumours and hematological malignancies (e.g. c-kit, 1p19q, t(9;22), HER-2/neu) involving cytogenetic and molecular genetic technologies (e.g., karyotyping, FISH, PCR, etc.). A multidisciplinary team developed 3 versions of a purpose-designed questionnaire for the 3 respondent groups (physicians, testing laboratories, referring laboratories), after finding no appropriate instrument in the literature. The questionnaire did not include validated items, though it was informed by questions asked of respondents in other relevant laboratory surveys[17,20-22]. It was pre-tested by the study team and 2–4 potential respondents per version using an iterative process to ensure face validity. The questionnaire was organized into 5 sections, 3 of which were similar across the 3 respondent groups: (i) the importance of MOT; (ii) training and continuing education for MOT; and (iii) demographic information. The other two sections addressed issues of specific concern to each respondent group, including questions (i) about the laboratory, and (ii) service provision, for laboratories, and (i) role in provision of MOT, and (ii) availability of MOT, for physicians. Correspondingly, the length of the questionnaire varied for each respondent group, with 150 items for physicians, 162 for referring laboratories and 195 items for testing laboratories.

We report data on the characteristics of physicians and laboratories and their reported involvement in MOT, together with responses in 6 key attitude domains: (i) **importance**: the perceived importance of MOT relative to other pathology or hematology testing for the diagnosis, prognosis or treatment of cancer, now and in 5 years; (ii) **adequacy of access**: perceptions regarding the adequacy of access by cancer patients to MOT within the respondent's region and Ontario, and in comparison to other jurisdictions in Canada or the US; (iii) **barriers to providing**: the perceived barriers to providing MOT, differentiated into those barriers seen to impede ordering MOT for physicians, and those barriers perceived to impede conducting MOT on-site, both for testing laboratories and any referring laboratories wishing to do so; (iv) **confidence in assessing indications**: reported confidence in assessing whether MOT was clinically indicated, and if so, *which *molecular oncology tests were indicated; in addition, because testing labs are expert providers of MOT and well-positioned to assess the relevance of such testing for particular types of cancers, we also asked testing laboratories to indicate their confidence in the ability of physicians and referring labs to make these determinations; (v) **responsibility of providers**: attitudes regarding which providers (physicians, referring laboratories, testing laboratory physician or institution as a whole) were perceived to appropriately determine which molecular oncology tests were suitable for individual patients, and for integrating the results of MO testing with other laboratory results; and (vi) **educational preparedness**: perceptions of how helpful various elements of formal or continuing education had been in preparing respondents to provide MOT on the one hand, and to maintain knowledge of MOT on the other. Additional items in the questionnaire addressed more detailed aspects of physician and laboratory practice in relation to MOT; a report of findings from these additional items is available, upon request, from the principal author.

Data from completed questionnaires were entered using the Snap Survey data entry tool (Version 8, Snap Surveys Ltd). As an exploratory study, our analysis measured only univariate associations. In the absence of clear prior hypotheses, we did not make any distributional assumptions, nor pursue multivariate modeling. Descriptive statistics were computed for all variables measured, including frequency counts and percentages. We used the chi-square test or, when appropriate, Fisher exact test to determine differences in categorical variables. Unadjusted odds ratios (ORs), 95% confidence intervals (CIs) and p-values are reported as appropriate. A probability level of < 0.05 was used to determine statistical significance. Physician data were analyzed using SPSS (Version 16). Laboratory data, and comparisons between laboratory and physician data, were analyzed using WinPepi COMPARE2 v1.78.

## Results

### Response rate

317 completed questionnaires were received from a total of 611 physicians contacted. During the course of the study, we identified 21 of the 611 physicians as ineligible and excluded them from the analysis giving us a corrected response rate of 53.73%. Reasons for exclusion included inability to locate physicians despite repeated efforts and respondents reporting themselves as ineligible. 57 completed laboratory questionnaires were received from the 99 laboratories contacted (17 of 30 testing labs, 40 of 69 referring labs). During the course of the survey, we identified 5 labs as ineligible as they did not assess cancer samples or conduct relevant testing, resulting in a corrected response rate of 60.64% (Table 1)

**Table 1:** Response rates

**Sample**	**Original No.**	**Completed surveys No.**	**Response rate** **(%)**	**Ineligible respondents No.**	**Adjusted response rate (%)**
Physicians	611	317	51.88	21*	53.73

					

Labs	99	57	57.58	5**	60.64

(Testing labs)	(30)	(17)	(56.67)	(3**)	(62.96)

(Referring labs)	(69)	(40)	(57.97)	(2**)	(59.70)

* Not in practice or could not be located despite repeated efforts.

** Lab did not assess cancer samples or conduct relevant testing.

### Characteristics and practices of respondents

Of the 317 completed physician questionnaires, 28 were excluded for poor quality or because physicians ordered no laboratory testing for cancer, leaving a total of 289 questionnaires for analysis (Table 2). The majority of these respondents were male (68.2%), worked in academic teaching unit settings (58.7%), in metropolitan/central city locations (59.1%), were medical specialists (81.7%), provided care only for patients with solid tumours (75.7%), and reported ordering any MOT (67.2%) (defined as rarely, sometimes, usually or always).

**Table 2:** Characteristics of physician respondents

**Characteristic**	**Respondents** **No. (%)**
**Practice setting**	

Academic teaching unit	162/276 (58.7)

Not-academic teaching unit	114/276 (41.3)

**Method of reimbursement**	

Fee for service	97/263 (36.9)

Not-fee for service	166/263 (63.1)

**Geographic location**	

Metropolitan/central city	165/279 (59.1)

Metropolitan/suburban	58/279 (20.8)

Small city or town	44/279 (15.8)

Rural	12/279 (4.3)

**Gender**	

Female	88/277 (31.8)

Male	189/277 (68.2)

**Primary area of practice or expertise**	

Specialty	219/268 (81.7)

Medical oncology	(81/268)

Radiation oncology	(77/268)

Surgical oncology	(24/268)

Hematology/Hematological oncology	(20/268)

Gynecological oncology	(9/268)

Medical and hematological oncology	(3/268)

Non-oncologic specialists (general internal medicine, gastroenterology)	(5/268)

Family medicine	49/268 (18.3)

**Type of oncology practice**	

Solid tumours only	212/280 (75.7)

Hematological malignancies only	12/280 (4.3)

Hematological malignancies and solid tumours	56/280 (20.0)

**Ordering Behaviour**	

Order any MOT	193/287 (67.2)

Order no MOT	94/287 (32.8)

Laboratories that performed MOT were more frequently located in central cities than referring labs (64.7% and 32.5% respectively); they more frequently served catchment areas with greater than 2,000,000 people (37.5% and 8.1% respectively), and were more frequently housed within academic health science centres (68.8% and 31.6% respectively) rather than community hospitals. In addition, more testing than referring laboratories defined themselves as molecular genetics (25.0% and 0% respectively) or cytogenetics (31.3% and 0% respectively) labs. These, and related, differences are statistically significant (Table 3).

**Table 3:** Characteristics of respondent laboratories

	**Testing Labs No. (%)**	**Referring Labs No. (%)**	**p-value**
**Geographic location**			

Metropolitan/central city	**11/17 (64.7)**	**13/40 (32.5)**	**0.024**

Metropolitan/suburban	4/17 (23.5)	9/40 (22.5)	1.000*

Small city/town	**2/17 (11.8)**	**16/40 (40.0)**	**0.036**

Rural	0/17 (0)	2/40 (5.0)	1.000*

**Size of catchment area**			

0 – 500,000	**2/16 (12.5)**	**21/37 (56.8)**	**0.003**

500,000 – 2,000,000	8/16 (50.0)	13/37 (35.1)	0.310

Greater than 2,000,000	**6/16 (37.5)**	**3/37 (8.1)**	0.016*

**Type of setting**			

Community hospital	**3/16 (18.8)**	**26/38 (68.4)**	**0.001**

Academic Health Science Centre	**11/16 (68.8)**	**12/38 (31.6)**	**0.012**

Research laboratory	2/16 (12.5)	0/38 (0)	0.084*

**Type of laboratory**			

General pathology	**1/16 (6.3)**	**22/38 (57.9)**	**< 0.001**

Anatomical pathology	1/16 (6.3)	9/38 (23.7)	0.249*

Hematopathology	2/16 (12.5)	0/38 (0)	0.084*

Hematology	1/16 (6.3)	3/38 (7.9)	1.000*

Cancer cytogenetics	1/16 (6.3)	0/38 (0)	1.000*

Cytology	0/16 (0)	2/38 (5.3)	1.000*

Flow cytometry	0/16 (0)	2/38 (5.3)	1.000*

Cytogenetics	**5/16 (31.3)**	**0/38 (0)**	0.001*

Molecular cancer genetics	1/16 (6.3)	0/38 (0)	0.296*

Molecular genetics	**4/16 (25.0)**	**0/38 (0)**	0.006*

* Fisher exact test

### Attitudes toward the importance of MOT

We asked respondents to indicate the importance of MOT relative to other pathology/hematology testing for the diagnosis, prognosis or treatment of cancer now, and in five years (using five-point *Likert *scales from not at all important to very important) (Table 4). Respondents shared a broadly positive opinion of the importance of MOT. Majorities of physicians, referring laboratories and testing laboratories considered MOT to be fairly or very important for treatment, diagnosis or prognosis now, and even larger majorities expressed these views for the importance of MOT in 5 years. Respondents differed, however, in the extent to which they deemed MOT to be *very *important. While the majority of testing labs judged MOT *very *important for treatment, prognosis and diagnosis now (70.6%, 64.7%, 76.5% respectively) and in 5 years (93.8%, 93.8%, 87.5% respectively), only a quarter to a half of physicians (24.6% through 52.2%) and referring labs (30.8% through 46.2%) had the same degree of enthusiasm; these differences are statistically significant.

**Table 4:** Perceived importance of MOT for diagnosis, prognosis, treatment now and in 5 years

	**Physicians No. (%)**		**Testing labs No. (%)**		**Referring labs No. (%)**		**p-value**
	Fairly important	Very important	Fairly important	Very Important	Fairly important	Very important	Very important

Treatment now	92/271 (33.9)	**85/271 (31.4)**	4/17 (23.5)	**12/17 (70.6)**	14/39 (35.9)	**15/39 (38.5)**	**0.004**

Prognosis now	127/273 (46.5)	**78/273 (28.6)**	5/17 (29.4)	**11/17 (64.7)**	13/39 (33.3)	**16/39 (41.0)**	**0.004**

Diagnosis now	98/272 (36.0)	**67/272 (24.6)**	3/17 (17.6)	**13/17 (76.5)**	13/39 (33.3)	**12/39 (30.8)**	**< 0.001**

							

Treatment in 5 yrs	79/270 (29.3)	**141/270 (52.2)**	1/16 (6.2)	**15/16 (93.8)**	14/39 (35.9)	**18/39 (46.2)**	**0.003**

Prognosis in 5 yrs	95/274 (34.7)	**140/274 (51.1)**	1/16 (6.2)	**15/16 (93.8)**	16/39 (41.0)	**18/39 (46.2)**	**0.003**

Diagnosis in 5 yrs	97/272 (35.7)	**109/272 (40.1)**	2/16 (12.5)	**14/16 (87.5)**	16/39 (41.0)	**15/39 (38.5)**	**0.001**

### Attitudes regarding access to MOT

We asked respondents to assess the adequacy of access by cancer patients to MOT within their region and in Ontario, and to compare access in Ontario to other regions in Canada and the US (using five-point *Likert *scales from strongly disagree to strongly agree) (Table 5). Responses did not differ significantly between groups. Attitudes were most positive regarding access in the respondent's own region, though only 45.3% of physicians, 58.8% of testing labs and 52.8% of referring labs mildly or strongly agreed that patients in their region were receiving MOT that is indicated as a standard of care. Even fewer respondents (31.2% to 41.2%) mildly or strongly agreed that cancer patients were receiving the standard of care for MOT in the province of Ontario as a whole; further, only 30.3% to 50% of respondents mildly or strongly agreed that Ontario compared favourably to other jurisdictions in Canada in ensuring clinically indicated MOT, while fewer still (6.7% to 14.2%) agreed that Ontario compared favourably with jurisdictions in the US in ensuring access to the standard of care in MOT.

**Table 5:** Perceived adequacy of access to MOT by cancer patients

	**Physicians No. (%)**		**Testing labs No. (%)**		**Referring labs No. (%)**		**p-value**
	Mildly agree	Strongly agree	Mildly agree	Strongly agree	Mildly agree	Strongly agree	Mildly or strongly agree

**Cancer patients are receiving the MOT that is indicated as a standard of care ...**							

...in my region	76/258 (29.5)	41/258 (15.9)	6/17 (35.3)	4/17 (23.5)	10/36 (27.8)	9/36 (25.0)	0.423

...in Ontario	58/253 (22.9)	21/253 (8.3)	3/17 (17.6)	4/17 (23.5)	10/37 (27.0)	4/37 (10.8)	0.535

**Ontario compares favorably in ensuring access to MOT that is indicated as a standard of care with...**							

... other jurisdictions in Canada	49/254 (19.3)	28/254 (11.0)	5/14 (35.7)	2/14 (24.3)	8/36 (22.2)	4/36 (11.1)	0.296

... other jurisdictions in the US	25/253 (9.9)	11/253 (4.3)	0/15 (0)	1/15 (6.7)	2/34 (5.9)	2/34 (5.9)	0.878*

*Fisher exact test

### Perceived barriers to MOT

To explore perceived barriers in the provision of MOT services, we identified a set of potentially relevant informational and material barriers and asked respondents to rate their impact (using five-point *Likert *scales from no impact to high impact) on their practice. Physicians were asked about barriers that might impede their ability or willingness to order MOT (Table 6). Laboratories, including both testing laboratories that already conducted MOT, and any referring laboratories that would like to provide MOT on-site, were asked about any barriers that might restrict their ability to provide MO testing (Table 7).

**Table 6:** Physician perception of barriers to ordering MOT

	**Some impact No. (%)**	**High impact No. (%)**
Lack of clear guidelines re. indications for MOT	82/261 (31.4)	100/261 (38.3)

Lack of Ontario Health Insurance Plan (OHIP) coverage	64/257 (24.9)	74/257 (28.8)

Lack of knowledge about how to order MOT	75/258 (29.1)	60/258 (23.3)

Lack of accessible lab services	77/257 (30.0)	49/257 (19.1)

Lack of knowledge about how to interpret MOT results	68/260 (26.2)	53/260 (20.4)

Lack of patient demand/interest	48/257 (18.7)	22/257 (8.6)

Lack of time or personnel to order or review MOT	43/258 (16.7)	17/258 (6.6)

**Table 7:** Laboratory perception of barriers to providing MOT (for testing labs and referring labs that wished to conduct MOT on-site)

	**Testing labs No. (%)**		**Referring labs@ No. (%)**		**p-value**
	Some impact	High impact	Some impact	High impact	High impact

Lack of funding for capital equipment	5/15 (33.3)	**6/15 (40.0)**	2/17 (11.8)	**14/17 (82.4)**	**0.014**

Lack of funding for technical staff	5/16 (31.2)	11/16 (68.8)	3/17 (17.6)	13/17 (76.5)	0.708*

Lack of funding for development of new tests (test work up)	6/16 (37.5)	10/16 (62.5)	4/17 (23.5)	12/17 (70.6)	0.622

Lack of funding for ongoing provision of tests	8/16 (50.0)	8/16 (50.0)	3/17 (17.6)	12/17 (70.6)	0.226

Lack of qualified technical staff	6/16 (37.5)	4/16 (25.0)	2/17 (11.8)	9/17 (56.3)	0.101

Lack of MDs and/or PhDs with appropriate expertise	6/16 (37.5)	3/16 (18.8)	3/17 (17.6)	6/17 (37.5)	0.438*

Lack of clinical demand	1/15 (6.7)	**6/15 (40.0)**	3/17 (17.6)	**1/17 (5.9)**	0.033*

Lack of appropriate test regulation and oversight	3/15 (20.0)	2/15 (13.3)	1/17 (5.9)	0/17 (0)	0.212*

Existence of restrictive patents or licenses	2/16 (12.5)	3/16 (18.8)	1/17 (5.9)	1/17 (5.9)	0.335*

@Referring labs were told to skip the question if they did not want to provide MOT on-site

*Fisher exact test

Among physicians, a lack of clear guidelines regarding clinical indications for MOT was perceived to be the most important barrier to ordering MOT (69.7% identified this as having some or high impact). Other barriers perceived to have some or high impact by a majority of physicians included a lack of coverage by the provincial health insurance plan (Ontario Health Insurance Plan, OHIP) (53.7%), and a lack of knowledge about how to order MOT (52.3%). Notably, only a quarter of physicians perceived a lack of patient demand, or a lack of time or personnel to order or review MOT, as barriers that posed some or a high impact (27.3% and 23.3% respectively).

Laboratory respondents perceived that most of the suggested resource barriers had some or a high impact on their practice, with the largest majorities perceiving the several items that identified potential funding barriers (e.g., lack of funding for technical staff, development of new tests, or ongoing provision of tests, etc.) as having some or high impact. Testing labs and those referring labs that did wish to provide MOT on-site were also similar in their perception of the two legal/regulatory barriers identified; only minorities of respondents perceived a lack of appropriate test regulation and oversight, or the existence of restrictive patents or licenses, as having some or high impact. However, testing and referring laboratories differed regarding some other potential barriers, with differences regarding which barriers had a high impact reaching statistical significance. For referring labs, the barrier of greatest concern was a perceived lack of funding for capital equipment: 82.4% of referring labs perceived this as a high impact barrier while only 40% of testing labs considered this a high impact barrier. Finally, the non-financial factor that attracted the most concern among testing labs was a perceived lack of clinical demand; significantly more testing labs than referring labs considered this a high impact barrier (40% and 5.9% respectively).

### Confidence in assessing indications for MOT

We asked respondents to specify their confidence in their ability to assess *whether *MO testing was clinically indicated or *which *MO tests were clinically indicated (using five-point *Likert *scales from not at all confident to very confident). Because testing labs occupy the end of the referral chain, we also asked testing labs to indicate their confidence in the ability of physicians and referring labs to make these determinations (Table 8).

**Table 8:** Stated confidence in assessing indications for MOT

	**Physicians about selves No. (%)**		**Referring labs about selves No. (%)**		**Testing labs about testing labs No. (%)**		**Testing labs about physicians No. (%)**		**Testing labs about referring labs No. (%)**	
	Fairly	Very	Fairly	Very	Fairly	Very	Fairly	Very	Fairly	Very

Whether MOT is indicated	102/276 (37.0)	25/276 (9.1)	21/39 (53.8)	3/39 (7.7)	8/17 (47.1)	5/17 (29.4)	11/17 (64.7)	1/17 (5.9)	1/17 (5.9)	0/17 (0)

										

Which MO tests are indicated	75/276 (27.2)	20/276 (7.2)	19/39 (48.7)	4/39 (10.3)	9/17 (52.9)	5/17 (29.4)	7/17 (41.2)	1/17 (5.9)	0/17 (0)	0/17 (0)

A large majority of testing labs was fairly or very confident of their ability to decide whether MOT was indicated (76.5%). By comparison, referring labs and physicians had less confidence in themselves in this domain (61.5% and 46% respectively were fairly or very confident). A majority of testing labs was also fairly or very confident of their ability to decide which MOT was required (82.4%); again, referring labs and physicians had less confidence regarding their own abilities (59% and 34.4% respectively were fairly or very confident). A majority of testing labs was fairly or very confident (70.6%) in the ability of physicians to determine *whether *MOT was required; but only a minority (5.9%) was fairly or very confident in referring labs to make this determination. Testing labs had less confidence in physicians to decide *which *MOT was indicated (47.1% were fairly or very confident) and *no *confidence (0% were fairly or very confident) in referring labs to make this determination.

### Preferred roles of clinicians and laboratories in managing MOT

The coordination of MO laboratory services involves the referring physician, the referring laboratory and two elements of the testing laboratory: the laboratory physician (i.e., hematologist/pathologist), and the laboratory as a reporting institution (i.e., not necessarily a physician registered to practice medicine in Ontario). To understand how respondents believed responsibilities should be shared among these stakeholders, we asked which parties should play a *determining *role in choosing the appropriate MOT and in integrating MOT results with other results to inform clinical practice (Table 9).

**Table 9:** Attitudes regarding who should play a determining role in MOT services

	**Physicians No. (%)^**	**Testing labs No. (%)^**	**Referring labs No (%)^**	**p-value**
**Choose appropriate MOT**				

Referring clinician	**195/269 (73.0)**	**5/16 (31.3)**	**21/38 (55.3)**	0.001*

Laboratory pathologist/hematologist	99/268 (37.1)	6/16 (37.5)	15/37 (40.5)	0.942

Referring laboratory	22/260 (8.5)	1/16 (6.3)	5/33 (15.2)	0.363*

Reporting laboratory	**15/258 (5.8)**	**8/16 (50.0)**	**9/33 (27.3)**	< 0.001*

**Integrate MOT results**				

Referring clinician	217/268 (81.3)	9/16 (56.3)	31/38 (81.6)	0.055

Laboratory pathologist/hematologist	**81/265 (30.6)**	**11/17 (64.7)**	**17/37 (45.9)**	**0.004**

Referring laboratory	**22/251 (8.8)**	**0/16 (0)**	**7/33 (21.2)**	0.043*

Reporting laboratory	**15/252 (6.0)**	**4/16 (25.0)**	**7/34 (20.6)**	0.001*

^Respondents could select more than one agent as having the 'determining role'; columns do not add to 100%

*Fisher exact test

In considering who should *choose *appropriate MOT, respondent groups did not differ significantly regarding the role of the referring laboratory or laboratory physician; few physicians, testing labs or referring labs indicated that the referring laboratory should play a determining role (8.5%, 6.3%, 15.2% respectively), while approximately one-third suggested that the laboratory physician should play a determining role (37.1%, 37.5%, 40.5% respectively). However, respondents did differ significantly regarding the relative roles of referring clinicians and testing laboratories. Physicians preferred that referring clinicians play a determining role (73%) and only 5.8% of physicians suggested this role for the testing laboratory. By contrast, 50% of testing labs preferred that testing labs play a determining role, while only 31.3% suggested that referring clinicians should have a determining role. Referring labs took an intermediate stance; 55.3% preferred that the referring clinician play a determining role and only 27.3% suggested that the testing lab should play a determining role in choosing appropriate MOT.

Attitudes regarding who should determine laboratory result *integration *showed a similar pattern. Majorities of respondent groups (56.3% of testing labs, 81.3% of physicians, 81.6% of referring labs) suggested that the referring clinicians should play a determining role. Yet 64.7% of testing laboratories preferred that laboratory physicians play a determining role – an option supported by only a minority of physicians (30.6%) and referring labs (45.9%). And while approximately one quarter of testing and referring laboratories (25%, 20.6% respectively) suggested that the testing lab should have a determining role, only 6% of physicians agreed.

### Reported educational preparedness for MOT

We asked respondents how helpful formal or continuing education had been in preparing them for, or allowing them to maintain their knowledge of, MOT (using five-point *Likert *scales from not at all helpful to very helpful) (Table 10). On balance, testing and referring laboratories reported being fairly well equipped by formal and continuing education to prepare for and maintain their knowledge of MOT, though respondents from testing and referring laboratories differed in terms of which formal educational resources had supported them. Respondents from testing laboratories relied on PhD training and post-PhD clinical training to equip them, and majorities reported these as fairly or helpful resources. Respondents from referring labs relied more heavily on undergraduate and post-graduate medical education, and most of these found such resources fairly or very helpful. Respondents from both testing and referring laboratories shared positive views regarding the helpfulness of most continuing educational resources, with majorities perceiving most identified resources as fairly or very helpful in maintaining knowledge of MOT.

By contrast, physicians were significantly less well served by their formal education; only 16.6% reported finding their undergraduate medical education fairly or very helpful and only 43.5% reported that their postgraduate medical education was fairly or very helpful. Further, significantly smaller majorities of physician than laboratory respondents found continuing educational resources helpful in enabling them to provide MO care; for example, only 67.6% of physicians compared to 94.1% testing labs and 97.1% of referring labs reported that CME was fairly or very helpful in maintaining knowledge of MOT.

**Table 10:** Perceived helpfulness of formal or continuing education for MOT

	**Physicians No. (%)**		**Testing labs No. (%)**		**Referring labs No. (%)**		**p-value**
	Fairly helpful	Very helpful	Fairly helpful	Very helpful	Fairly helpful	Very helpful	Fairly or very helpful

**Helpfulness of formal education in preparation for MOT**							

Undergraduate medical education	**37/265 (14.0)**	**7/265 (2.6)**	**2/5 (40.0)**	**3/5 (60.0)**	**10/34 (29.4)**	**7/34 (20.6)**	< 0.001*

Postgraduate medical education	**77/253 (30.4)**	**33/253 (13.0)**	**1/4 (25.0)**	**2/4 (50.0)**	**16/33 (48.5)**	**13/33 (39.4)**	< 0.001*

Master's degree (e.g., MSc, MA)	16/88 (18.2)	6/88 (6.8)	0/3 (0)	0/3 (0)	2/9 (22.2)	2/9 (22.2)	0.362*

Doctoral degree (e.g., PhD, DPhil)	**7/65 (10.8)**	**12/65 (18.5)**	**5/15 (33.3)**	**6/15 (40.0)**	**2/7 (28.6)**	**3/7 (42.9)**	0.001*

Post-PhD clinical training	N/A	N/A	2/13 (15.4)	11/13 (84.6)	4/7 (57.1)	2/7 (28.6)	0.350*

**Helpfulness of continuing education resources in maintaining knowledge of MOT**							

Discussions with colleagues	**120/264 (45.5)**	**79/264 (29.9)**	**5/17 (29.4)**	**11/17 (64.7)**	**18/35 (51.4)**	**14/35 (40.0)**	0.023*

Conferences, workshops, meetings	**122/264 (46.2)**	**73/264 (27.7)**	**6/17 (35.3)**	**10/17 (58.8)**	**17/38 (44.7)**	**17/38 (44.7)**	0.001*

Reading journal articles	**127/267 (47.6)**	**59/267 (22.1)**	**5/17 (29.4)**	**11/17 (64.7)**	**20/35 (57.1)**	**14/35 (40.0)**	< 0.001*

Continuing medical education (CME)	**120/262 (45.8)**	**57/262 (21.8)**	**8/17 (47.1)**	**8/17(47.1)**	**15/34 (44.1)**	**18/34 (52.9)**	**< 0.001**

Electronic resources (websites, listservs, etc.)	**86/258 (33.3)**	**35/258 (13.6)**	**8/17 (47.1)**	**7/17 (41.2)**	**18/35 (51.4)**	**8/35 (22.9)**	**< 0.001**

In-service training	N/A	N/A	4/10 (40.0)	2/10 (20.0)	8/22 (36.4)	5/22 (22.7)	1.000*

*Fisher exact test

### Associations between attitudes and physician characteristics

We explored whether physicians' responses to questions across the 6 attitude domains reviewed above were associated with characteristics that we judged might improve a physician's ability to make effective use of MOT services, including whether the physician: (i) reported having *ever *ordered MO testing; (ii) was a specialist rather than a family physician; (iii) provided *any *care for patients with hematological malignancies; (iv) practiced within an academic teaching unit setting; (v) worked in a metropolitan area (city or suburb) rather than a small town or rural area; and (vi) was a recent graduate (graduating between 1998–2007) compared to a less recent graduate (1958 – 1997) (see Additional file 1).

Of the 36 items tested in these univariate analyses of association, the characteristic of having ever ordered MOT was consistently associated (statistically significant for 24 of 36 items) with more positive attitudes and the perception of fewer barriers. Specifically, physicians who reported ever having ordered MOT were more likely than those who had never ordered MOT to: (a) consider it very important for diagnosis, prognosis and treatment now, and in 5 years, (b) mildly or strongly agree that cancer patients were receiving the MOT that is indicated as a standard of care in their region or Ontario, and that Ontario compares favourably in ensuring access in this regard with other jurisdictions in Canada or the US; (c) be fairly or very confident of their ability to assess whether and which MOT was indicated; and (d) perceive their formal and informal education as fairly or very helpful in allowing them to prepare for and maintain knowledge of MOT. Finally, physicians who reported ever having ordered MOT were less likely than those who had never ordered MOT to (e) perceive identified factors as barriers that had some or a high impact on their ability to order MOT (the sole exception was in being *more *likely to perceive the lack of provincial coverage as a barrier).

Two other physician characteristics – being a specialist (18/36 statistically significant associations) or providing any care for patients with hematological malignancies (11/36 statistically significant associations) – were typically but not uniformly associated with more positive attitudes and the perception of fewer barriers, as reviewed above. The final two physician characteristics – working in a metropolitan area (4/36 statistically significant associations) and being a recent graduate (1/36 statistically significant associations) – were only infrequently associated with more positive attitudes and the perception of fewer barriers.

## Discussion

Clinical molecular oncology testing (MOT) for the acquired genomic alterations underlying sporadic cancer has an increasingly important role to play in the diagnosis and treatment of cancer. Basic science research to identify relevant biomarkers is increasing[27], but health services research to support the translation of discovery research into effective clinical care is lacking[1,6]. Molecular oncology testing is complex, and likely to be done in those few facilities with the requisite technical and professional expertise[28]; thus, effective delivery requires coordinated systems of referral and reporting to ensure that relevant testing is completed, clear reports are provided to treating clinicians, and clinicians are empowered to use this information in appropriate ways to guide clinical practice. In Ontario, Canada, policy makers have begun to take an interest in the development of effective molecular oncology services[29], but little research is available to support decision-making. We report the results of a postal survey of the three key stakeholder groups involved in the effective provision of these testing services – physicians involved in cancer care, laboratories where MOT is conducted on-site (i.e., testing laboratories) and laboratories that receive cancer samples but do not conduct MOT on-site (i.e., referring laboratories) – to identify attitudes and reported practices, provide a baseline data set, and suggest areas of concern for future hypothesis-driven research.

Our results suggest that physicians, testing laboratories and referring laboratories shared positive attitudes regarding the importance of MOT for diagnosis, prognosis and treatment now and expected it to be still more important in 5 years. Testing labs were, however, significantly more likely to consider MOT *very *important than the physicians and referring labs that ensure appropriate referral. Further, despite general support for the value of MOT, respondents shared a dim view of access to needed molecular oncology services. They were most enthusiastic about access within their own region – approximately half of our respondents mildly or strongly agreed that cancer patients were receiving the MOT that was indicated as a standard of care in their region. Smaller proportions agreed that the standard of care was being met in the province as a whole, or that Ontario compared favourably to other jurisdictions in Canada or the US in ensuring access. Further, respondents perceived various barriers to ordering or providing MOT. Physicians perceived several barriers to ordering MOT, notably a lack of clear guidelines regarding the clinical indications for MOT, and a lack of adequate coverage by the province's health insurance plan. Testing laboratories that currently provided MOT on-site, and those referring laboratories that wished to do so, emphasized the impact of lack of funding on their ability to provide MOT, with majorities indicating that a lack of funding for technical staff, capital equipment (especially for referring labs), test work up and ongoing provision of MOT services posed some or high impact. Of note, only small minorities of physicians and referring labs perceived a lack of clinical demand as a barrier, but almost half of all testing labs perceived it as such.

These reported results identify perceptions rather than objective reality, and the appropriateness of reported attitudes and their impact on practice is unknown. Nonetheless, these results do identify some issues for further research. In particular, the limited agreement among respondents with the suggestion that patients were receiving access to clinically indicated MOT is worrisome. In addition, the fact that testing labs deem MOT more important than the physicians and referring labs who must ensure that samples are referred appropriately, together with the relatively greater perception among testing labs of reduced patient demand, may point to impediments in referral that could restrict patient access.

Concern about the ability of stakeholders to ensure the appropriate provision of MOT is enhanced by significant differences in confidence and the perceived helpfulness of education among respondent groups. Testing labs were well equipped by formal and continuing education and were confident about providing MOT. Yet referring labs, while equally well equipped by educational resources, were considerably less confident than testing labs in their ability to determine whether and which MOT was indicated, nor did they attract the confidence of testing labs. Physicians reported being even less well prepared. They were especially poorly equipped by their formal education and expressed limited confidence in their ability to decide whether MOT was indicated or what tests to order. This may suggest that physicians and referring labs are less able to make appropriate referrals for MOT than would be advisable. Finally, despite physicians' lack of self-confidence, they wished to maintain control over the selection and interpretation of MOT. By contrast, testing labs perceived a determining role for themselves in selecting appropriate MOT and for the laboratory physician to integrate MOT with other laboratory results to guide clinical practice. These differences suggest a lack of clarity about responsibility for the coordination and use of appropriate MO services.

The pattern of associations between physician characteristics and reported attitudes and perceived barriers is not conclusive, but raises questions about whether the ordering behaviour of physicians is appropriate. Physicians who ordered any MOT, specialists, physicians who provided any care for patients with hematological malignancies, and physicians in academic teaching unit settings were more likely than their peers (i.e., those who ordered no MOT, family physicians, those who did not provide hematological care, etc.) to report greater enthusiasm for the importance of MOT, better access to needed testing, more confidence to provide MOT and the greater helpfulness of formal and continuing education. Other physician characteristics (e.g., metropolitan workplace, recent graduates) were less consistently important but still significant factors influencing more positive attitudes and experiences. Some of these differences were expected, reflecting the more established importance of MOT for the hematological malignancies and the greater responsibility of specialist than family physician providers to ensure adequate MOT for patients in their care. Further research will be required to determine whether these statistically significant differences reflect appropriate patterns of practice or suggest that ordering behaviour is driven by *other *factors than clinical rationale.

Surveys reporting the attitudes and practices of laboratories or physicians in genetic testing for hereditary disease [17-19], or in molecular genetic testing more generally[20,21], have been reported, but this survey is a first to assess the role of physicians and laboratories in ensuring appropriate access to MOT for the sporadic cancers. Expanded knowledge of clinically important genomic biomarkers increases the contribution of the clinical laboratory to overall patient care[30], and increases the need for effective laboratory-clinician communication[31]. Arguably, it also increases the need for vertically integrated labs that can standardize the selection of appropriate testing, including MOT[28], and increases the need to "place responsibility for primary interpretation of laboratory tests on laboratory professionals"[4]. This study suggests that laboratories and physicians in Ontario may not be well equipped as individual stakeholders, or as a coordinated system of referral and interpretation, to provide MOT. Deficits in education, knowledge and confidence, and in the ability to coordinate appropriate testing, together with perceived funding shortfalls, may compromise optimal cancer care.

The strengths of this survey include its novelty, detailed questionnaire and rigorous follow-up to ensure a relatively high response rate. Limitations result from a sampling frame for physicians that relied on self-identification by hematologists of a sub-specialty or medical interest in oncology for inclusion. This is likely to have reduced the representation of eligible hematology respondents and may have biased the results for physicians toward a more negative view of access to, or preparedness for, MOT. In addition, the research reported here fails to reflect the rapid pace of recent developments in molecular oncology that have reinforced the value of MOT for the hematological malignancies and demonstrated its value for more of the solid tumours. The biasing effect of this limitation is likely to be similar to that of a reduced representation of hematological respondents, producing a less positive view of the value of MOT than might be apparent if the survey were to be run again today. Further, data about each laboratory were provided by one respondent, who could only partially represent the features of organizational practice and culture that are likely to be relevant to effective laboratory operation. Finally, this research uses self-report, rather than objective, data on MOT use, and our results are limited by the use of multiple tests of statistical significance on the same data. Further research is needed to address these limitations and to identify educational, training and other interventions to rectify deficits in care.

## Conclusion

Physicians and laboratory professionals reported being enthusiastic about the value of MOT for cancer care but many did not believe that patients in their care were gaining adequate access to clinically necessary testing. Further, our results suggest that many respondents were ill equipped as individual stakeholders, or as a coordinated system of referral and interpretation, to provide MOT. These challenges, together with perceived funding shortfalls, should inspire educational, training and other interventions to ensure that developments in molecular oncology can result in optimal cancer care.

## Competing interests

The authors declare that they have no competing interests.

## Authors' contributions

FM led the study, coordinated the development of the questionnaire, directed the data analysis, and drafted and revised the manuscript. PK assisted with the design of the questionnaire and the conduct of the study and provided direction for the statistical analysis. RJC assisted with the development of the questionnaire and the conduct of the survey and performed the data analysis. CA assisted with the development of the questionnaire, coordinated the conduct of the survey and assisted with the data analysis. RFC and SKR played essential advisory roles in the development of the survey instrument and the analysis of the data. All authors read and approved the final manuscript.

## Authors' information

RFC and SKR lead key labs in Ontario that conduct molecular oncology testing. In addition, both have led policy initiatives through the Ontario Ministry of Health and Long-Term Care and Cancer Care Ontario to garner attention for this area of laboratory practice and cancer care.

## Pre-publication history

The pre-publication history for this paper can be accessed here:



## Supplementary Material

Additional file 1 Statistically significant associations between key physician characteristics and physician attitudes regarding MOT. Click here for file
