# Supplementary material for: Postal survey of physicians and laboratories: Practices and perceptions of molecular oncology testing
Source: BMC Health Serv Res. 2009 Jul 30;9:131. doi: 10.1186/1472-6963-9-131 (PMC2731034; doi:10.1186/1472-6963-9-131)
Supplement: Additional file 1 — Statistically significant associations between key physician characteristics and physician attitudes regarding MOT. [file 1472-6963-9-131-S1.doc]

Additional file 1. Statistically significant associations between key physician characteristics and physician attitudes regarding MOT

| **Association with considering MOT very important now** | **Very important**  **No. (%)** | **Not very important**  **No. (%)** | **Unadjusted OR (95% CI)** | **p-value** |
| --- | --- | --- | --- | --- |
| Diagnosis Now | | | | |
| Order any MOT | 61 (32.97) | 124 (67.03) | 6.48 (2.67 - 15.69) | < 0.001 |
| Order no MOT | 6 (7.06) | 79 (92.94) | 1.00 |
| Specialist physician | 59 (27.96) | 152 (72.04) | 3.03 (1.14 - 8.05) | 0.021 |
| Family physician | 5 (11.36) | 39 (88.64) | 1.00 |
| Hematology provider | 29 (43.94) | 37 (56.06) | 3.47 (1.90 - 6.32) | < 0.001 |
| Non-hematology provider | 38 (18.45) | 168 (81.55) | 1.00 |
| **Prognosis Now** | | | | |
| Order any MOT | 65 (34.95) | 121 (65.05) | 3.27 (1.65 - 6.46) | < 0.001 |
| Order no MOT | 12 (14.12) | 73 (85.88) | 1.00 |
| Hematology provider | 32 (47.76) | 35 (52.24) | 3.18 (1.78 - 5.68) | < 0.001 |
| Non-hematology provider | 46 (22.33) | 160 (77.67) | 1.00 |
| Treatment Now | | | | |
| Order any MOT | 70 (37.63) | 116 (62.37) | 2.74 (1.45 - 5.15) | 0.001 |
| Order no MOT | 15 (18.07) | 68 (81.93) | 1.00 |
| Hematology provider | 30 (45.45) | 36 (54.55) | 2.27 (1.28 - 4.04) | 0.005 |
| Non-hematology provider | 55 (26.83) | 150 (73.17) | 1.00 |
| **Association with considering MOT very important in 5 years** | **Very important**  **No. (%)** | **Not very important**  **No. (%)** | **Unadjusted OR (95% CI)** | **p-value** |
| Diagnosis in 5 Years | | | | |
| Order any MOT | 94 (50.81) | 15 (17.65) | 4.82 (2.57 - 9.03) | < 0.001 |
| Order no MOT | 91 (49.19) | 70 (82.35) | 1.00 |
| Specialist physician | 91 (43.33) | 10 (22.73) | 2.60 (1.22 - 5.54) | 0.011 |
| Family physician | 119 (56.67) | 34 (81.27) | 1.00 |
| Hematology provider | 40 (60.61) | 69 (33.50) | 3.06 (1.72 - 5.41) | < 0.001 |
| Non-hematology provider | 26 (39.39) | 137 (66.50) | 1.00 |
| Prognosis in 5 Years | | | | |
| Order any MOT | 112 (59.89) | 28 (32.94) | 3.04 (1.77 - 5.21) | < 0.001 |
| Order no MOT | 75 (41.11) | 57 (67.06) | 1.00 |
| Hematology provider | 48 (71.64) | 92 (44.44) | 3.16 (1.74 - 5.74) | < 0.001 |
| Non-hematology provider | 19 (28.36) | 115 (55.56) | 1.00 |
| Treatment in 5 Years | | | | |
| Order any MOT | 116 (62.70) | 25 (30.12) | 3.90 (2.24 - 6.80) | < 0.001 |
| Order no MOT | 69 (37.30) | 58 (69.88) | 1.00 |
| Specialist physician | 118 (56.46) | 14 (32.56) | 2.69 (1.34 - 5.38) | 0.004 |
| Family physician | 91 (43.54) | 29 (67.44) | 1.00 |
| Hematology provider | 45 (69.23) | 96 (46.83) | 2.56 (1.41 - 4.63) | 0.002 |
| Non-hematology provider | 20 (30.77) | 109 (53.17) | 1.00 |
| **Association with agreeing (mildly or strongly) regarding access to MOT** | **Mildly or strongly agree No. (%)** | **Not mildly or strongly agree No. (%)** | **Unadjusted OR**  **(95% CI)** | **p-value** |
| Cancer patients are receiving the MOT that is indicated as a standard of care ... in my region | | | | |
| Order any MOT | 101 (55.8) | 80 (44.2) | 4.73 (2.54 - 8.84) | < 0.001 |
| Order no MOT | 16 (21.05) | 60 (78.95) | 1.00 |
| Specialist physician | 101 (50) | 101 (50) | 3.75 (1.64 - 8.58) | 0.001 |
| Family physician | 8 (21.05) | 30 (78.95) | 1.00 |
| Hematology provider | 39 (58.21) | 28 (41.79) | 2.02 (1.15 - 3.55) | 0.014 |
| Non-hematology provider | 78 (40.84) | 113 (59.16) | 1.00 |
| Academic teaching unit | 77 (50.99) | 74 (49.01) | 1.87 (1.11 – 3.16) | 0.018 |
| Non-academic teaching unit | 35 (35.71) | 63 (64.29) | 1.00 |
| Cancer patients are receiving the MOT that is indicated as a standard of care ... in Ontario | | | | |
| Order any MOT | 69 (38.98) | 108 (61.02) | 4.15 (2.00- 8.63) | < 0.001 |
| Order no MOT | 10 (13.33) | 65 (86.67) | 1.00 |
| Specialist physician | 69 (35.03) | 128 (64.97) | 3.56 (1.33 - 9.50) | 0.008 |
| Family physician | 5 (13.16) | 33 (86.84) | 1.00 |
| Association with agreeing (mildly or strongly) regarding access to MOT | **Mildly or strongly agree No. (%)** | **Not mildly or strongly agree No. (%)** | **Unadjusted OR**  **(95% CI)** | **p-value** |
| Ontario compares favorably in ensuring access to MOT that is indicated as a standard of care with ... other jurisdictions in Canada | | | | |
| Order any MOT | 66 (36.87) | 113 (63.13) | 3.35 (1.65 - 6.80) | 0.001 |
| Order no MOT | 11 (14.86) | 63 (85.14) | 1.00 |
| Specialist physician | 69 (34.67) | 130 (65.33) | 4.379 (1.49 – 12.87) | 0.004 |
| Family physician | 4 (10.81) | 33 (89.19) | 1.00 |
| Academic teaching unit | 54 (36.49) | 94 (63.51) | 2.18 (1.20 - 3.96) | 0.009 |
| Non-academic teaching unit | 20 (20.83) | 76 (79.17) | 1.00 |
| **Ontario compares favorably in ensuring access to MOT that is indicated as a standard of care with ... other jurisdictions in the US** | | | | |
| Order any MOT | 32 (18.08) | 145 (81.92) | 3.92 (1.33 - 11.51) | 0.008 |
| Order no MOT | 4 (5.33) | 71 (94.67) | 1.00 |
| Recent graduates (1998-2007) | 7 (43.75) | 9 (56.25) | 5.29 (1.82, 15.44) | 0.013 |
| Less recent graduates (1958-1997) | 26 (12.81) | 177 (87.19) | 1.00 |
| **Association with perceiving factors as barriers (some or high impact)** | **Some or high impact No. (%)** | **Not some or high impact No. (%)** | **Unadjusted OR (95% CI)** | **p-value** |
| **Lack of clear guidelines about indications for MOT** | | | | |
| Order any MOT | 117 (65.36) | 62 (34.64) | 0.50 (0.27 - 0.93) | 0.027 |
| Order no MOT | 64 (79.01) | 17 (20.99) | 1.00 |
| Lack of knowledge how to order MOT | | | | |
| Order any MOT | 83 (46.63) | 95 (53.37) | 0.48 (0.28 - 0.83) | 0.008 |
| Order no MOT | 51 (64.56) | 28 (35.44) | 1.00 |
| Specialist physician | 95 (46.8) | 108 (53.2) | 0.19 (0.08 - 0.46) | < 0.001 |
| Family physician | 32 (82.05) | 7 (17.95) | 1.00 |
| Hematology provider | 27 (41.54) | 38 (58.46) | 0.56 (0.32 - 0.99) | 0.044 |
| Non-hematology provider | 108 (55.96) | 85 (44.04) | 1.00 |
| Academic teaching unit | 67 (45.27) | 81 (54.73) | 0.43 (0.26 - 0.73) | 0.001 |
| Non-academic teaching unit | 67 (65.69) | 35 (34.31) | 1.00 |
| Metro city / suburban | 102 (49.76) | 103 (50.24) | 0.511 (0.26 - 0.99) | 0.045 |
| Small town/ rural | 31 (65.96) | 16 (34.04) | 1.00 |
| Lack of knowledge how to interpret MOT | | | | |
| Order any MOT | 69 (38.76) | 109 (61.24) | 0.37 (0.22 - 0.64) | < 0.001 |
| Order no MOT | 51 (62.96) | 30 (37.04) | 1.00 |
| Specialist physician | 84 (41.18) | 120 (58.82) | 0.30 (0.14 – 0.62) | 0.001 |
| Family physician | 28 (70) | 12 (30) | 1.00 |
| Lack of patient demand/ interest | | | | |
| Order any MOT | 30 (16.95) | 147 (83.05) | 0.21 (0.12 – 0.38) | < 0.001 |
| Order no MOT | 39 (49.37) | 40 (50.63) | 1.00 |
| Specialist physician | 46 (22.77) | 156 (77.23) | 0.25 (0.12 - 0.51) | < 0.001 |
| Family physician | 21 (53.85) | 18 (46.15) | 1.00 |
| Hematology provider | 9 (9.47) | 86 (90.53) | 0.35 (0.16 - 0.74) | 0.005 |
| Non-hematology provider | 61 (31.77) | 131 (68.23) | 1.00 |
| Academic teaching unit | 33 (22.15) | 116 (77.85) | 0.506 (0.29 – 0.89) | 0.017 |
| Non-academic teaching unit | 36 (36) | 64 (64) | 1.00 |
| **Lack of Ontario Health Insurance Plan (OHIP) Coverage** | | | | |
| Order any MOT | 103 (58.19) | 74 (41.81) | 1.84 (1.08 – 3.15) | 0.025 |
| Order no MOT | 34 (43.04) | 45 (56.96) | 1.00 |
| **Association with confidence (fairly or very confident) to assess indications for MOT** | Fairly or very confident  **No. (%)** | **Not fairly or very confident**  **No. (%)** | **Unadjusted OR (95% CI)** | **p-value** |
| Whether MOT indicated | | | | |
| Order any MOT | 110 (60.11) | 73 (39.89) | 7.06 (3.82 - 13.07) | < 0.001 |
| Order no MOT | 16 (17.58) | 75 (82.42) | 1.00 |
| Specialist physician | 121 (56.02) | 95 (43.98) | 58.59 (7.94 - 432.6) | < 0.001 |
| Family physician | 1 (2.13) | 46 (97.87) | 1.00 |
| Hematology provider | 39 (58.21) | 28 (41.79) | 1.92 (1.10 - 3.35) | 0.021 |
| Non-hematology provider | 88 (42.11) | 121 (57.89) | 1.00 |
| Academic teaching unit | 88 (55.7) | 70 (44.3) | 2.62 (1.57 - 4.37) | < 0.001 |
| Non-academic teaching unit | 35 (32.41) | 73 (67.59) | 1.00 |
| Which MOT indicated | | | | |
| Order any MOT | 86 (46.99) | 97 (53.01) | 8.07 (3.83 – 17.05) | < 0.001 |
| Order no MOT | 9 (9.89) | 82 (90.11) | 1.00 |
| Specialist physician | 89 (41.2) | 127 (58.8) | 66.69 (4.18 to 1064.58)* | < 0.001 |
| Family physician | 0 (0) | 47 (100) | 1.00 |
| Academic teaching unit | 29 (43.28) | 38 (56.72) | 3.59 (2.02 – 6.40) | < 0.001 |
| Non-academic teaching unit | 66 (31.58) | 143 (68.42) | 1.00 |
| **Association with perceiving formal education as helpful (fairly or very) in preparation of MOT** | **Fairly or very helpful**  **No. (%)** | **Not fairly or very helpful**  **No. (%)** | **Unadjusted OR**  **(95% CI)** | **p-value** |
| Undergraduate medical education | | | | |
| Order any MOT | 37 (20.22) | 146 (79.78) | 3.04 (1.30 - 7.12) | 0.008 |
| Order no MOT | 7 (7.69) | 84 (92.31) | 1.00 |
| Specialist physician | 38 (17.76) | 176 (82.24) | 4.86 (1.13 - 20.90) | 0.020 |
| Family physician | 2 (4.26) | 45 (95.74) | 1.00 |
| Academic teaching unit | 33 (21.02) | 124 (78.98) | 2.64 (1.24 - 5.61) | 0.010 |
| Non-academic teaching unit | 10 (9.17) | 99 (90.83) | 1.00 |
| Postgraduate medical education | | | | |
| Order any MOT | 95 (52.2) | 87 (47.8) | 5.39 (2.88 - 10.08) | < 0.001 |
| Order no MOT | 15 (16.85) | 74 (83.15) | 1.00 |
| Specialist physician | 99 (46.26) | 115 (53.74) | 6.89 (2.62 - 18.13) | < 0.001 |
| Family physician | 5 (11.11) | 40 (88.89) | 1.00 |
| Hematology provider | 36 (53.73) | 31 (46.27) | 2.07 (1.19 - 3.62) | 0.010 |
| Non-hematology provider | 74 (35.92) | 132 (64.08) | 1.00 |
| **Association with perceiving continuing education as helpful (fairly or very) in maintaining knowledge of MOT** | **Fairly or very helpful**  **No. (%)** | **Not fairly or very helpful**  **No. (%)** | **Unadjusted OR**  **(95% CI)** | **p-value** |
| Continuing medical education | | | | |
| Order any MOT | 141 (77.05) | 42 (22.95) | 4.94 (2.86 - 8.53) | < 0.001 |
| Order no MOT | 36 (40.45) | 53 (59.55) | 1.00 |
| Specialist physician | 149 (69.63) | 65 (30.37) | 4.16 (2.11 - 8.17) | < 0.001 |
| Family physician | 16 (35.56) | 29 (64.44) | 1.00 |
| Metro city / suburban | 146 (67.28) | 71 (32.72) | 2.06 (1.10 - 3.83) | 0.022 |
| Small town / rural | 25 (50) | 25 (50) | 1.00 |
| Conferences, workshops | | | | |
| Order any MOT | 150 (81.52) | 34 (18.48) | 4.72 (2.70 – 8.25) | < 0.001 |
| Order no MOT | 43 (48.31) | 46 (51.69) | 1.00 |
| Specialist physician | 165 (76.74) | 50 (23.26) | 4.13 (2.12 – 8.04) | < 0.001 |
| Family physician | 20 (44.44) | 25 (55.56) | 1.00 |
| Academic teaching unit | 121 (76.1) | 38 (23.9) | 1.93 (1.13 – 3.30) | 0.015 |
| Non-academic teaching unit | 66 (62.26) | 40 (37.74) | 1.00 |
| Metro city / suburban | 161 (73.52) | 58 (26.48) | 2.08 (1.10 – 3.95) | 0.023 |
| Small town/ rural | 28 (57.14) | 21 (42.86) | 1.00 |
| Discussions with colleagues | | | | |
| Order any MOT | 148 (80.87) | 35 (19.13) | 3.28 (1.87- 5.75) | < 0.001 |
| Order no MOT | 49 (56.32) | 38 (43.68) | 1.00 |
| Specialist physician | 168 (78.87) | 45 (21.13) | 4.10 (2.08 – 8.05) | < 0.001 |
| Family physician | 21 (47.73) | 23 (52.27) | 1.00 |
| Academic teaching unit | 126 (80.25) | 31 (19.75) | 2.50 (1.43 – 4.36) | 0.001 |
| Non-academic teaching unit | 65 (61.9) | 40 (38.1) | 1.00 |
| Reading journal articles | | | | |
| Order any MOT | 147 (79.89) | 37 (20.11) | 4.99 (2.87 - 8.69) | < 0.001 |
| Order no MOT | 39 (44.32) | 49 (55.68) | 1.00 |
| Specialist physician | 162 (75.35) | 53 (24.65) | 7.29 (3.56 - 14.95) | < 0.001 |
| Family physician | 13 (29.55) | 31 (70.45) | 1.00 |
| Academic teaching unit | 120 (75.47) | 39 (24.53) | 2.40 (1.41 - 4.07) | 0.001 |
| Non-academic teaching unit | 59 (56.19) | 46 (43.81) | 1.00 |
| Metro city / suburban | 160 (73.39) | 58 (26.61) | 3.68 (1.94 - 6.98) | < 0.001 |
| Small Town / rural | 21 (42.86) | 28 (57.14) | 1.00 |
| **Electronic resources** | | | | |
| Order any MOT | 97 (53.3) | 85 (46.7) | 2.95 (1.69 - 5.13) | < 0.001 |
| Order no MOT | 24 (27.91) | 62 (72.09) | 1.00 |
| Specialist physician | 103 (48.58) | 109 (51.42) | 2.14 (1.06 - 4.33) | 0.032 |
| Family physician | 13 (30.23) | 30 (69.77) | 1.00 |

*Where cells are empty (i.e., a sampling zero), 0.5 was added to each cell to calculate the confidence interval, as recommended by Agresti A: *An introduction to categorical data analysis*. Second edition. New York: John Wiley; 2007.
